# The Coexistence of Chronic Obstructive Pulmonary Disease and Heart Failure

**DOI:** 10.7759/cureus.17387

**Published:** 2021-08-23

**Authors:** Khizer Khalid, Jaskamal Padda, Anton Komissarov, Lanson B Colaco, Sandeep Padda, Armughan S Khan, Victor Melt Campos, Gutteridge Jean-Charles

**Affiliations:** 1 Internal Medicine, Jean-Charles (JC) Medical Center, Orlando, USA; 2 Internal Medicine, Avalon University School of Medicine, Willemstad, CUW; 3 Internal Medicine, Advent Health & Orlando Health Hospital, Orlando, USA

**Keywords:** copd, heart failure, bnp, nt-pro bnp, echocardiogram, pft

## Abstract

Chronic obstructive pulmonary disease (COPD) is a chronic illness that is widely prevalent within the United States and has been frequently associated with heart failure (HF). COPD is associated with progressive damage and inflammation of the airways leading to airflow obstruction and inadequate gas exchange. HF represents a decline in the normal functioning of the heart resulting in insufficient pumping of blood through the circulatory system. COPD and HF present with similar signs and symptoms with some variation.

There are many specific diagnostic tests and treatment modalities which we use to diagnose COPD and HF, but it becomes an issue when you come across a patient who has both conditions simultaneously. For example, attempting to use an X-ray to diagnose HF in a COPD patient is next to impossible because the results are manipulated by the COPD disease process. This is the case with many other diagnostic tests such as an electrocardiogram (ECG), chest radiography (X-ray), B-type natriuretic peptide (BNP), echocardiogram, cardiac magnetic resonance imaging (CMR), pulmonary function test (PFT), arterial blood gas (ABG), and exercise stress testing.

When a patient has both COPD and HF, it becomes more difficult to treat. Many treatments for HF have negative impacts on COPD patients and vice-versa, whereas some have also shown positive clinical outcomes in both diseases. It is agreeable that treatment has to be patient-centered and it can vary from case to case depending on the severity of the disease. Ultimately, in this review, we discuss COPD and HF and how they interplay in their diagnostic and treatment modalities to gain a better understanding of how to effectively manage patients who have been diagnosed with both conditions.

## Introduction and background

Chronic obstructive pulmonary disease (COPD) is among the top four chronic illnesses prevalent in the United States and the fifth most common disease globally [1]. Cardiovascular diseases such as heart failure (HF) have been observed to be frequently associated with COPD. The general chief complaint in both diseases is dyspnea [2,3]. HF represents a decline in the normal functioning of the heart. The heart is unable to pump a sufficient amount of blood into the circulatory system. This can be due to either an abnormality in the heart’s contractile apparatus or due to an inability to fill the left ventricle. The disease can either present acutely or progress slowly. COPD occurs due to progressive damage and inflammation of the bronchi. This leads to a limited outflow of air from the lungs and an inadequate gas exchange [4]. From this information, we can deduce that both diseases have similar presentations despite different pathophysiologies [3]. COPD and CHF also share similar risk factors such as advanced age, systemic inflammation, tobacco smoking and obesity [5], also both groups may present with similar comorbidities such as malignancy, sleep apnea, etc [6]. Therefore, it is no surprise that there is a great deal of overlap in clinical practice and both diseases often exist simultaneously. All this complicates the process of diagnosing and prescribing treatment to patients with either one or both diseases [3]. This review aims to go through the pathophysiology, diagnosis, and treatment of COPD and CHF as well as exploring some of the problems that clinicians encounter while dealing with patients suffering from these ailments.

## Review

Pathophysiology of COPD and HF

COPD is defined as “a common, preventable, and treatable disease that is characterized by persistent respiratory symptoms and airflow limitation that is due to airway and/or alveolar abnormalities usually caused by significant exposure to noxious particles or gases and influenced by host factors including abnormal lung development” [6]. It's a chronic pulmonary illness with a slow progression and systemic consequences [7]. The American Heart Association defines HF as “a complex clinical syndrome that can result from any structural or functional cardiac disorder that impairs the ability of the ventricle to fill with or eject blood”. In this disease process, as cardiac output decreases, many compensatory pathways are activated which in turn increases the blood pressure and blood volume. The efforts of these pathways eventually lead to further myocardial damage and worsening contractility. HF still remains a clinical diagnosis based on patient medical history, findings on the physical exam, and laboratory testing. Symptoms of HF include fatigue, shortness of breath (SOB), and signs of volume overload such as peripheral edema [8].

The interplay of pathophysiologic processes of the respiratory and cardiovascular systems in individuals with COPD and HF is complicated and modified by the effect of pharmacologic medications used to treat both illnesses. Moreover, trials have shown that the possible side effects associated with the use of medications to treat COPD might result in cardiovascular complications and those used for HF management might worsen COPD features [9,10]. This is observed in the usage of beta 2-agonists in the treatment of respiratory disorders and beta-blockers (BB) in the treatment of cardiovascular disorders [11-14]. Smoking serves as a common risk factor for both COPD and HF. It is important to note that COPD in itself is a risk factor in the development of HF [15,16]. Both systolic and diastolic dysfunction of the right and left ventricles are seen in patients with COPD [17,18]. The right HF results as a consequence of hypoxic vasoconstriction of lung-induced pulmonary hypertension [19]. Right HF eventually causes Left HF [20,21]. 

There may be systemic inflammation present, which might play a role in the various comorbid illnesses seen in COPD patients [22]. This chronic systemic inflammation involves both innate and adaptive immunity and affects the parenchyma as well as the bronchial walls [23]. Similarly, cardiovascular diseases are associated with low-grade systemic inflammation [24,25]. Airflow obstruction as a result of the bronchial wall thickening, has been linked to a number of cardiovascular risk factors such as exaggerated subclinical atherosclerosis and both endothelium-dependent and endothelium-independent vasodilation [26-28]. Furthermore, although not consistently, C-reactive protein levels have been linked to structural and functional changes in the vascular system of COPD patients [26,29-31]. It can thus be summarised that individuals with COPD have an increased risk of developing HF as they share similar risk factors and pathophysiologic processes.

Signs and symptoms

COPD and HF tend to present with similar signs and symptoms. Those which are common to both include exertional SOB, paroxysmal nocturnal dyspnea (PNH), nocturnal cough, fatigue, depression, and anxiety [32,33]. Patients with COPD generally present with symptoms of dyspnea, cough with/without sputum production, and a history of tobacco smoking [34]. Conversely, one should suspect HF in COPD patients, who have symptoms of orthopnea, nocturnal cough, PNH, acute pulmonary edema, easy fatigability, and reduced exercise tolerance in the absence of a chest infection [33]. 

The cardiac and pulmonary physical examinations of patients with coexistence of COPD and HF are generally normal because of the presence of lung hyperinflation. Crackles may be perceived due to the opening of small airways in COPD. Wheezing is generally heard due to the restriction of airflow in the smaller airways in CHF patients, whereas crackles of pulmonary edema may not be heard in a hyperinflated torso. Indications of ankle edema, jugular venous distention, and hepatomegaly in COPD patients indicate the notion of right ventricular malfunction, and with the occurrence of a loud P2 and left parasternal heave the implication of cor pulmonale can be made. Ultimately, it is not enough to have physical exam findings to diagnose COPD and HF, additional testing needs to be performed to arrive at the correct diagnosis [33].

Diagnostic tests of COPD and HF

Electrocardiogram (ECG)

It has been proven that the ECG has an outstanding negative predictive value which is able to practically dismiss chances of chronic HF secondary to left ventricular systolic dysfunction [35]. Nonetheless, it is not a practice that is widely used to diagnose HF especially when there are many abnormalities present, such as in patients who have COPD and HF in conjunction [5].

Chest Radiography (X-ray)

Chest radiography is unable to detect the coexistence of HF and COPD mainly because it is manipulated by the presence of COPD. Chest hyperinflation tends to decrease the cardiothoracic ratio, and the heart becomes long and narrow in appearance. Pulmonary edema is also concealed due to the radiolucent lung fields and pulmonary vascular remodeling. Chest radiography may not be useful in HF and COPD, but it may be suitable to detect other diseases [32,33].

BNP

In COPD patients, levels of natriuretic peptides are a rapid and sensitive biomarker to identify HF. Cardiac myocytes secrete BNP and N-terminal fragment of B-type natriuretic peptide (NT-proBNP) in response to increased atrial and ventricular filling pressure [5]. When BNP levels are below 100 pg/mL, this effectively excludes HF. In COPD patients, when BNP levels range from 100 pg/mL to 500 pg/mL it indicates either cor pulmonale as a cause, left ventricular failure, or even both [5]. It has also been suggested that BNP levels >500 pg/mL in COPD patients do not distinguish between a cardiac or pulmonary cause for deterioration, but it does indicate that HF treatment should be started or upgraded along with treatment for COPD [5,33,36].

A study conducted by Rutten et al. evaluated BNP levels and the risk of HF in 200 elderly patients with COPD. Four natriuretic peptide assays were able to rule out HF, but the positive predictive value and the accuracy were less than what was observed in the patients with acute dyspnea. The results indicated that the stable patients had lower levels of BNP compared to those who had increased intracardiac pressures and acute volume overload. It was also determined that BNP levels are generally increased in patients with COPD [37]. The National Institute for Health and Care Excellence (NICE) guidelines indicate HF as a diagnosis if BNP levels are >400 pg/mL or if NT-proBNP levels are >2000 pg/mL, and if there is still skepticism it is recommended to conduct cardiac imaging studies [5]. Furthermore, once patients who have had COPD exacerbations return to normal baseline, imaging of the heart is required in order to provide suitable treatment based on the results [36].

Echocardiogram

Performing an echocardiogram in patients with COPD can uncover left ventricular dysfunction which can be related to the manifestation of cardiovascular disease. An echocardiographic assessment of the right ventricle is also important to evaluate the presence of cor pulmonale in patients with COPD and to determine their prognosis [5]. The NICE guidelines state that if BNP >400 pg/mL or NT-proBNP >2000 pg/mL, an echocardiogram is recommended within two weeks and if BNP is 100 pg/mL to 400 pg/mL or NT-proBNP is 400 pg/mL to 2000 pg/mL, it is recommended within six weeks. HF would be ruled out if the echocardiogram is normal, whereas if the ejection fraction (EF) >40% with irregular left ventricular mass or the presence of an enlarged left atrium, HF should still be kept in mind in regard to COPD patients. If and when the left ventricular EF reaches <40% in COPD patients, it is recommended to initiate full HF therapy [5,33].

Roughly 20% to 25% of HF patients have BNP levels <100 pg/mL, which is why echocardiography is a more dependable test to identify left ventricular systolic dysfunction in stable COPD patients [36]. While echocardiography is an important diagnostic test for HF, it can be limited in patients who are obese, and the imaging can be obstructed due to poor acoustic windows in COPD patients who have pulmonary hyperinflation. In such situations, it may be necessary to utilize other diagnostic tools such as magnetic resonance imaging or radionuclide ventriculography [5,32,36].

Cardiac Magnetic Resonance Imaging (CMR)

CMR is used to measure left ventricular volumes and ejection fraction. It allows for exact measures of right ventricular volume, flow, and function. The outcome of the test is known to be reproducible, accurate, and widely validated. It is able to interpret tissue characterization in order to identify myocardial fibrosis that can lead to arrhythmias. It is widely recommended to use CMR to evaluate the function of the left ventricle in patients with HF whose echocardiogram images are limited [32].

Pulmonary Function Test (PFT)

PFT results are generally described in an obstructive or restrictive manner. In emphysema and COPD patients an obstructive pattern is observed, whereas in HF, more of a restrictive pattern is displayed. Thus, when a patient has both COPD and HF, they can display both obstructive and restrictive configurations on the PFT [5]. Objective confirmation of airflow obstruction is required to make the diagnosis of COPD. The Global Initiative for Chronic Obstructive Lung Disease (GOLD) criteria states that in order to make a diagnosis of COPD, the post-dilatory ratio of forced expiratory volume in one second and forced vital capacity needs to be less than 0.7 (FEV1/FVC <0.7), using spirometry (Table 1) [6]. Ultimately, if HF patients are treated appropriately, lung function can improve. It is advised to repeat spirometry testing after HF treatment in order to make a final assessment of whether the patient has COPD [5,33].

**Table 1:** Classification of airflow limitation severity in COPD (based on post-bronchodilator FEV1). FEV1: forced expiratory volume in one second; FVC: forced vital capacity. Reproduced with permission. © 2020, Global Initiative for Chronic Obstructive Lung Disease, available from www.goldcopd.org, published in Fontana, WI, USA [6].

In patients with FEV_1_/FVC < 0.70:		
GOLD 1	Mild	FEV_1_ ≥ 80% predicted
GOLD 2	Moderate	50% ≤ FEV_1_ < 80% predicted
GOLD 3	Severe	30% ≤ FEV_1_ < 50% predicted
GOLD 4	Very severe	FEV_1_ < 30% predicted

Arterial Blood Gas (ABG)

The ABG is used to detect the variations of gas exchange in patients with COPD and HF. HF essentially worsens the process of gas exchange whereas COPD decreases the O2 arterial pressure and increases the arterial pressure of CO2. This in turn results in hypoxemia [5].

Exercise Stress Test

Cardiopulmonary exercise testing has shown considerably weak responses from patients with COPD and HF. There is a significantly worse response in patients who have both diseases which are proven with a few elements such as minute ventilation/carbon dioxide production slope, peak oxygen consumption, and heart rate recovery at one minute [5]. A study was conducted by Guazzi et al. in order to interpret the combined impact of COPD and HF on the exercise stress test. A total of 69 patients with both HF and COPD and 69 patients with only HF were selected for the study. They all underwent pretesting with PFT, echocardiography, doppler imaging, and then cardiopulmonary exercise testing (CPX). It was eventually determined that patients with both HF and COPD have appreciably reduced response to CPX and this will have an influence on the clinical interpretation of the CPX data in patients with both conditions [38].

Influences of HF management in COPD

Beta-Blockers

It is a well-known fact that patients with HF are under-prescribed with BB if there is a COPD comorbidity for fear of potential bronchial spasm [39]. A Cochrane Library meta-analysis study showed that cardioselective beta1-blockers (B1Bs) in COPD patients provided no significant change in FEV1 or respiratory symptoms compared to placebo and did not affect the FEV1 treatment response to beta2-agonists [2]. It included 22 randomized, blinded, controlled trials (-2.55% of FEV1 [CI, 5.94 to 0.84], P = 0.4 for studies of longer duration). It concluded that the use of B1Bs is safe in patients with COPD [40].

Mtisi, et al. analysed several observational studies that showed a mortality benefit of B1Bs in HF patients with coexistent COPD [41]. One retrospective study using the Taiwan National Health Insurance Research Database evaluated the effects of carvedilol, bisoprolol, and metoprolol in 11,558 COPD & HF comorbid patients. When compared with control, bisoprolol use was related to a significant dose-dependent mortality benefit [low-dose bisoprolol HR = 0.76 (95% CI, 1.47-1.85); high-dose bisoprolol hazard ratio (HR) = 0.40 (95% confidence interval (CI), 0.26-0.63), P <0.001] [42]. There is also evidence of a better clinical outcome in COPD patients, who are prescribed BBs, either with comorbid HF or not [43].

Diuretics

Diuretics are an important part of the symptomatic therapy of fluid retention in HF. But due to COPD comorbidity, they can potentially cause several side effects, as cardiac output decreases, venous return reduction, hypokalemia, hypercapnia, and metabolic alkalosis [44], thus, increasing risks of complications in COPD patients. On the other hand, diuretics might bring benefits to COPD therapy by alleviating lung congestion and reducing the work of breathing [45-47]. So, it is crucial to titrate the dose of diuretics according to the patient's symptoms to relieve fluid retention and dyspnea while preserving cardiac output and venous return.

Angiotensin-Converting Enzyme Inhibitors/Aldosterone Receptor Blockers

Blockage of the Renin-Angiotensin-Aldosterone System (RAS system) remains a cornerstone of CHF treatment, as it is a vital part of its pathophysiology. Angiotensin-converting enzyme (ACE) is expressed mainly in the pulmonary endothelium; thus, the lungs are particularly susceptible to the effects of circulating RAS products. Regular use of ACE inhibitors does not increase the bronchospasm risk, nor the risk of cough or angioedema in COPD patients [48]. Moreover, some retrospective studies have shown ACE inhibitors and ARBs have been associated with slower emphysema progression in smokers and FEV1 decline in COPD [49-51]. However, the OPTIMIZE-HF cohort study showed COPD patients with comorbid HF are less likely to be prescribed with ACE inhibitors or ARBs during their hospital stay [52].

Hydralazine and Other Vasodilators

Vasodilators, such as hydralazine or nitrates, reduce heart afterload and preload. However, it negatively affects cor pulmonale, which develops in COPD patients [53]. This type of medication induces pulmonary vasodilation of poorly ventilated areas leading to hypoxemia [54,55]. Because of it, when prescribed to HF-COPD patients, arterial partial oxygen pressure should be tightly monitored.

Ivabradine

Ivabradine is a novel anti-tachycardic drug that is used in HF patients. It has been established that tachycardia is a predictor of mortality in both HF or COPD patients. The SHIFT trial showed that Ivabradine was equally safe and effective in reducing mortality or hospitalization in HF-COPD and HF alone patients [56].

Influences of COPD management in HF

Clinical guidelines do not recommend altering COPD treatment with coexisting HF [1]. It can be challenging for clinicians to treat COPD with comorbid HF. This is because mainstay therapies used for COPD have been known to have adverse cardiovascular profiles [57].

Beta-2 Agonists

Inhaled beta-2 agonists are frequently used to treat COPD. Due to their non-selective nature, these drugs can increase heart rate causing an increased myocardial oxygen demand. This side effect occurs in both oral and inhaled routes of administration. There has been a study reporting a worsening of HF and an increased rate of hospitalization with the use of these drugs [58]. The Study to Understand Mortality and Morbidity in COPD (SUMMIT) was a four-armed double-blinded randomized controlled trial that studied the relation of beta-2 agonist (vilanterol) with cardiovascular disease. Participants were COPD patients with cardiovascular disease, and they were randomly (1:1:1:1) assigned treatment with vilanterol alone, vilanterol plus inhaled fluticasone furoate, fluticasone furoate alone, or placebo. Outcomes of the study did not show an increase in all-cause mortality or exacerbation in cardiovascular disease with the use of beta-agonist therapy alone and neither when used in combination [9]. Accordingly, the study claims that beta-2 agonist therapy can be deemed safe in patients with cardiovascular disease. More recent studies have also shown that the use of bronchodilator therapy (beta-2 agonists and anticholinergic agents) can somewhat alter the left ventricular stiffness seen in patients with COPD. These studies were done on a small scale for short time intervals and included inadequate study samples, so generalization of such findings to all HF patients is inappropriate [18,59,60]. One flaw with the SUMMIT study was the exclusion of patients with low ejection fractions (left ventricular ejection fraction <30%) and patients with implantable cardiac defibrillators (ICD). Therefore, hesitance in prescribing beta-2 agonists persists in clinicians dealing with COPD and HF [3].

Inhaled Steroids

Other medications used in managing COPD include inhaled or oral steroids. Oral steroids cause an increase in salt and water retention. This can augment the volume overload in HF. This effect is dose-dependent and higher doses (>20 mg/day prednisone) carry a greater risk of aggravating congestive failure. As a result, oral steroid use is discouraged. Inhaled steroids are comparatively more favorable and can be used when indicated [5].

Methylxanthines

Methylxanthines such as theophylline are contraindicated as they carry a high risk of causing arrhythmias [57]. Roflumilast, a phosphodiesterase-four inhibitor, has not shown to be harmful to HF patients [61,62].

Anticholinergics

Anticholinergic agents are largely considered safe and are employed as alternate bronchodilators in patients with cardiovascular disease. The safety profile appears to be consistent throughout the drug class [57]. Although their effects on HF are under-studied when compared to other bronchodilators such as beta-2 agonists [63].

Antibiotics

Antibiotics such as macrolides are used to treat COPD exacerbations. Due to the QT-prolonging side effect, their use can be considered less favorable for HF patients who are already vulnerable to arrhythmias [64].

Non-invasive ventilation (NIV) in COPD and HF

Cranston et al. have stated that long-term oxygen therapy (>15 hours per day) has been demonstrated to improve survival in patients with chronic respiratory failure who have significant resting hypoxemia [65]. However, a randomized controlled trial done in 2016 concluded that this therapy did not prolong time to death or initial hospitalization, nor did it give a sustained benefit for any of the evaluated outcomes in individuals with stable COPD and resting or exercise-induced mild arterial oxygen desaturation [66]. NIV works by increasing oxygenation and decreases the mechanical effort of breathing [67]. Patients with hypercapnic respiratory failure secondary to exacerbation of COPD or with HF in the settings of acute pulmonary edema benefit from NIV in addition to pharmacologic treatment. 

When compared to standard oxygen therapy, NIV improves gas exchange and symptoms in COPD patients, lowering the requirement for endotracheal intubation, hospital mortality, and hospital stay. NIV may also reduce the amount of time under invasive mechanical ventilation and further avoid reintubation. NIV expedites the remission of symptoms, normalization of blood gas values in acute cardiogenic pulmonary edema, lowers the necessity for endotracheal intubation, and is associated with a decreased mortality [68]. Biphasic Positive Airway Pressure (BiPAP) can help individuals with cor pulmonale caused by COPD and improve their right ventricular function [69]. It has also been identified to have a significant role in the treatment of HF patients who have developed muscle fatigue and hypercapnia [70,71].

NIV may be especially beneficial in situations of acute respiratory failure of mixed origin (COPD with pulmonary edema) because it may help both underlying diseases [72]. In patients hospitalized with an acute exacerbation of COPD and acute respiratory failure, NIV in the form of non-invasive positive pressure ventilation has been established as the standard of treatment for reducing both morbidity and mortality [73-76]. In all patients with pulmonary edema as a consequence of HF and acute HF associated with COPD, NIV is recommended as first-line therapy [77]. NIV has been shown to reduce the frequency of the need for endotracheal intubation and mechanical ventilation, resulting in higher survival, lower complication rates, and shorter intensive care unit and hospital admission in patients with COPD [78,79].

## Conclusions

Patients with COPD or CHF have many interlocking signs and symptoms such as dyspnea, nocturnal cough, fatigue, wheezing, crackles on auscultation etc. Thus, proper diagnosis of both conditions is crucial for appropriate management. Many diagnostic tests are available for the diagnosis of COPD and HF. The challenge occurs when these conditions occur simultaneously, leading to one disease process interfering with the others diagnostic tests. Further investigation is required to find a more effective way to make the correct diagnosis in order to provide adequate care for the patient. Simultaneous management of COPD and HF remains quite an arduous task. Clinicians should rely on their acumen, past experiences, and available evidence while prescribing treatment to patients. Treatment must be patient-centered and can vary from case to case depending on associated comorbidities and their severity. Further research is required to clarify guidelines and to study the interactions of the various drugs used to diagnose and treat COPD and HF.
